# Unexpected Normal Colloid Osmotic Pressure in Clinical States with Low Serum Albumin

**DOI:** 10.1371/journal.pone.0159839

**Published:** 2016-07-25

**Authors:** Regina Michelis, Shifra Sela, Teuta Zeitun, Ronit Geron, Batya Kristal

**Affiliations:** 1 Eliachar Research Laboratory, Galilee Medical Center, Nahariya, Israel; 2 Nephrology Department, Galilee Medical Center, Nahariya, Israel; 3 Faculty of Medicine in the Galilee, Bar Ilan University, Safed, Israel; University Medical Center Utrecht, NETHERLANDS

## Abstract

**Background:**

In clinical states associated with systemic oxidative stress (OS) and inflammation such as chronic kidney disease (CKD), oxidative modifications of serum albumin impair its quantification, resulting in apparent hypoalbuminemia. As the maintenance of oncotic pressure/colloid osmotic pressure (COP) is a major function of albumin, this study examined the impact of albumin oxidation on COP, both in-vivo and in-vitro.

**Methods:**

Patients with proteinuria and patients on chronic hemodialysis (HD) with systemic inflammation and OS were enrolled. Blood samples were collected from 134 subjects: 32 healthy controls (HC), proteinuric patients with high (n = 17) and low (n = 31) systemic inflammation and from 54 patients on chronic hemodialysis (HD) with the highest levels of OS and inflammation.

**Results:**

In-vitro oxidized albumin showed significantly higher COP values than non-oxidized albumin at identical albumin levels. In vivo, in hypoalbuminemic HD patients with the highest OS and inflammation, COP values were also higher than expected for the low albumin levels. The contribution to COP by other prevalent plasma proteins, such as fibrinogen and immunoglobulins was negligible.

We imply that the calculation of COP based on albumin levels should be revisited in face of OS and inflammation. Hence, in hypoalbuminemic proteinuric patients with systemic OS and inflammation the assumption of low COP should be verified by its measurements.

## Introduction

Albumin is the most abundant protein in human plasma with remarkably diverse functions including antioxidant activity, buffering properties, binding and transport capacities for numerous substances (free fatty acids, various ions, NO, bilirubin, peptides, uremic toxins and drugs). Physiologically, maintenance of oncotic pressure/colloid osmotic pressure (COP) is considered its major function as it controls the distribution of extracellular fluid between the vascular and extra-vascular compartments [1,2]. Albumin is predominantly an interstitial protein with only 40% of its total amount in the intravascular fluid [1–3]. Although albumin accounts for 50–60% of the plasma protein mass, it provides 75–80% of COP, due to its relatively low molecular mass (~67KDa, van’t Hoff law) [1]. Its negative charge also plays a role in COP maintenance, by attracting cations such as sodium (Na^+^) and causing water molecules to shift across the semi-permeable capillary membrane into the intravascular space (Gibbs-Donnan effect) [1].

The steady state concentration of albumin in plasma depends on the rates of biosynthesis and degradation, and on its inter-compartmental distribution. Hypoalbuminemia (albumin <3.8 g/dl) is prevalent in clinical states associated with chronic inflammation and severe oxidative stress (OS), such as in patients on hemodialysis therapy (HD) [4,5]. In agreement with the established increase in OS in chronic kidney disease (CKD) patients [6], recent studies have shown that apparent hypoalbuminemia is partially due to oxidation of albumin which impairs its quantification by the standard laboratory assay using bromocresol-green (BCG) [4,7]. Specifically, with this assay, the hypoalbuminemia observed in proteinuric and HD patients results partially from impaired detection of modified/oxidized albumin molecules [7].

In pathologic conditions such as CKD or proteinuria, reactive oxygen species (ROS) that normally play important roles in normal cellular physiology are involved in various injurious consequences such as systemic inflammation and protein modifications. The present study evaluated the impact of albumin oxidation on measurements of COP in proteinuric patients with various degrees of systemic inflammation and of hypoalbuminemia.

## Materials and Methods

All chemicals and antibodies were obtained from SIGMA (St. Louis, MO, USA), unless specified otherwise.

### Subjects

Blood was drawn from 134 subjects: healthy controls (HC, n = 32), proteinuric patients with low (n = 31) or high (n = 17) degrees of systemic inflammation as revealed by normal (<5 mg/l) or high C-reactive protein (>5 mg/l, CRP) levels (proteinuria low inflammation and proteinuria high inflammation groups), and an HD group (n = 54, chronic HD therapy, 4 hours of treatment thrice weekly, for at least one year). Within our proteinuric patient cohort 48% had non nephrotic range proteinuria and 44% had nephrotic syndrome. Sixty two percent of the nephrotic syndrome patients were CKD, i.e. had GFR < 60ml/min/1.73m2. All patients and HC subjects had normal liver function and no evidence of infection or malignancy. Blood of HD patients was drawn from the arterial line before dialysis. Sera were separated immediately and frozen at -70°C. The study was approved by the Helsinki Committee (Institutional Review Board) of Galilee Medical Center, Nahariya, Israel, in compliance with the declaration of Helsinki, and all subjects signed a written informed consent form.

### Determination of "albumin detection index" in sera

Serum albumin was isolated by gel-filtration (GF) chromatography as described previously [4,7]. The albumin-detection index is defined as the ratio of the BCG read-out (clinical assay) to the total albumin concentration, as determined by OD_280_ [4] in the albumin-containing fractions. The BCG assay was performed according to the Aeroset chemical analyzer instructions (ABBOTT laboratories, USA).

### Measurement of advanced oxidation protein products in sera

Levels of advanced oxidation protein products (AOPP), a marker of protein oxidation, were measured in sera as described previously [8].

### Determination of inflammatory cytokines in serum

Systemic inflammation was assessed in sera by the levels of CRP (by Immunoturbidimetry using the Architect/Aeroset analysers ABBOTT Laboratories), and interleukin-6 (IL-6) and TNFα (by ELISA Quantikine, R&D systems).

### Oncotic pressure measurements

COP was measured using a colloid osmometer (Wescor, Logan, USA), which operates using a membrane with a cutoff of 30kDa; thus the measured oncotic pressure relates only to proteins with a molecular mass >30kDa. The minimal sample volume for determination of COP is 0.5ml.

### In-vitro preparation of AGE-albumin, AOPP-albumin and unmodified albumin

Commercial, pure human serum albumin (HSA), further purified on VIVASPIN columns (Sartorius Stedium biothech Gmbh, Germany, cutoff 30kDa), was used as unmodified HSA. For *in-vitro* oxidation of HSA, this unmodified HSA was incubated with glucose or with 50mM HOCl (Aldrich) as described [4,9]. Control HSA samples were incubated identically, but without glucose or HOCl. Non-proteinaceous contents were removed by dialysis overnight (membrane cutoff 12kDa). The resulting AGE-HSA and AOPP-HSA preparations were used immediately, without storage.

## Purification of serum albumin

For albumin purification from sera, Cibacron Blue 3GA Agarose (CB3GA) was inserted into a SigmaPrep spin column and washed four times by additions of 10mM Tris pH 8.0 and short centrifugations (8,000g, 10s). Serum (150μl) was added to the washed CB3GA, incubated at r.t. for 10 min and centrifuged (12,000g, 1 min). This step was repeated. The twice depleted serum was discarded and elution buffer (150μl, 10mM Tris pH 8.0 and 1.5M NaCl) was added to the column. After 10 min incubation the albumin samples were eluted by centrifugation (12,000g, 1 min) into a new collection tube. To deplete immunoglobulin contaminations, 100μl of the eluted albumin sample were incubated (with shaking) for 3hrs at 4°C with 20μl (washed and diluted into 900μl H_2_O) of protein A/G Ultralink Resin (Thermo Scientific, USA). The resin bound immunoglobulins were discarded by centrifugation. The supernatant was concentrated by SPEEDVAC centrifugation (approx. 3h).

### Partial depletion of albumin in HC sera

To study the effect of HC and HD albumin on COP, serum albumin levels had to be comparable. As BCG-measured albumin levels in patients were lower than in HC, COP values could not be compared between these groups in the hypoalbuminemic range of albumin levels. Therefore, albumin levels had to be decreased artificially in samples of HC sera, prior to COP measurement. Twenty HC sera were partially albumin-depleted using the same CB3GA procedure, except that the depletion step was performed only once and the column bound albumin was discarded. The obtained partially-albumin-depleted HC sera (resulting albumin levels: 1.9–2.9 g/dl) were used for further analyses.

### Oncotic pressure measurements in purified albumin from sera

Samples of purified albumin from sera, were concentrated (by SPEEDVAC) to a final concentration of 5mg/100μl. The colloid osmometer poses two technical limitations: it requires relatively high protein concentrations and large sample volumes (~0.5ml). Purification from sera yields albumin amounts that cannot meet these requirements. Therefore, equal volume of each purified albumin sample was added to 300μl of normal (HC) sample with known albumin level and index (a single HC serum was used in each experiment). This step increased both the albumin level and COP value in each sample. The Δ in COP could then be calculated per the Δ in albumin level to be expressed as the COP conferred by 1g/dl of albumin.

### Statistical analysis

Data parameters were analyzed by unpaired t-test, by linear regression analysis and by Wilcoxon Signed Ranks Test, as appropriate. P<0.05 was considered significant. The mean±SE are shown unless where SD is specified.

## Results

### Characteristics of study population

The characteristics of HC subjects and patients are given in Table 1. Albumin and COP values were decreased in patients, while systemic inflammation and OS were increased, as evident by CRP and OS markers (Table 1).

**Table 1:** Characteristics of the study population.

		**HC**	**proteinuria low inflammation**	**proteinuria high inflammation**	**HD**
	**n**	32	31	17	54
	**Gender (male/female)**	12/20	17/14	7/10	28/23
	**Age (Years)**	45±2*	55.6±2.8	64.7±3.6	62±3
	**% of diabetics**	-	39	59	53
	**Serum Albumin (g/dl)**	3.9±0.06*	3.2±0.1	3.0±0.2	3.0±0.1
	**COP**	28.8±0.4*	21.0±0.9	21.9±1.1	24.5±0.6*
**Oxidative stress markers**	**Albumin detection Index**	0.93±0.03*	0.78±0.04^#^	0.66±0.06^#^	0.53±0.03*
	**AOPP (μmol/l)**	67±4*	92±7*	113±10*	188±9*
**Inflammatory markers**	**CRP (mg/l)**	3.2±1.0^a^	3.6±0.4^a^	46.0±10.1 ^b^	29.9±7.7 ^b^
	**TNFα (pg/ml)**	[0.55–2.816]^§^	7.4±0.9	11.6±2.2	nd
	**IL-6 (pg/ml)**	[1.1–14.3]^§^	2.3±0.4	7.4±1.9	nd
**Kidney function parameters**	**eGFR (ml/min/1.73m**^**2**^**)** ^**c**^ **[range]**	nd	53±6 [8–131]	38±8 [8–120]	nd
	**Urinary protein excretion (g/24h), [range]**	nd	5.4±0.6 [0.3–13.7]	5.0±1.1 [1.1–17.7]	nd
	**Serum Creatinine (mg/dl)[range]**	nd	2.0±0.3 [0.5–5.6]	2.7±0.4 [0.6–5.5]	nd

All values are given as mean±SE.

* indicates significant p value (p<0.05) compared to each of the other groups.

^#^ indicates significant p value compared with the HC and HD groups.

^a^ indicates significant p value compared with each one of the groups: HD and proteinuria with high inflammation.

^b^ indicates significant p value compared with each one of the groups: HC and proteinuria with low inflammation. "nd" Not determined.

^**c**^ Estimated glomerular filtration rate (eGFR) was calculated by the MDRD equation

§ Data not available, normal range is given.

The reliability of the BCG assay for albumin quantification is markedly impaired by oxidation of albumin resulting in underestimation of its concentration [4,7]. This was also manifested in the index values of the HD group, that showed a decrease of >40% vs. HC, as well as 20% and 32% decreases vs. proteinuria with high and low degrees of inflammation, respectively (Table 1).

## Albumin levels and COP values

**Fig 1:**
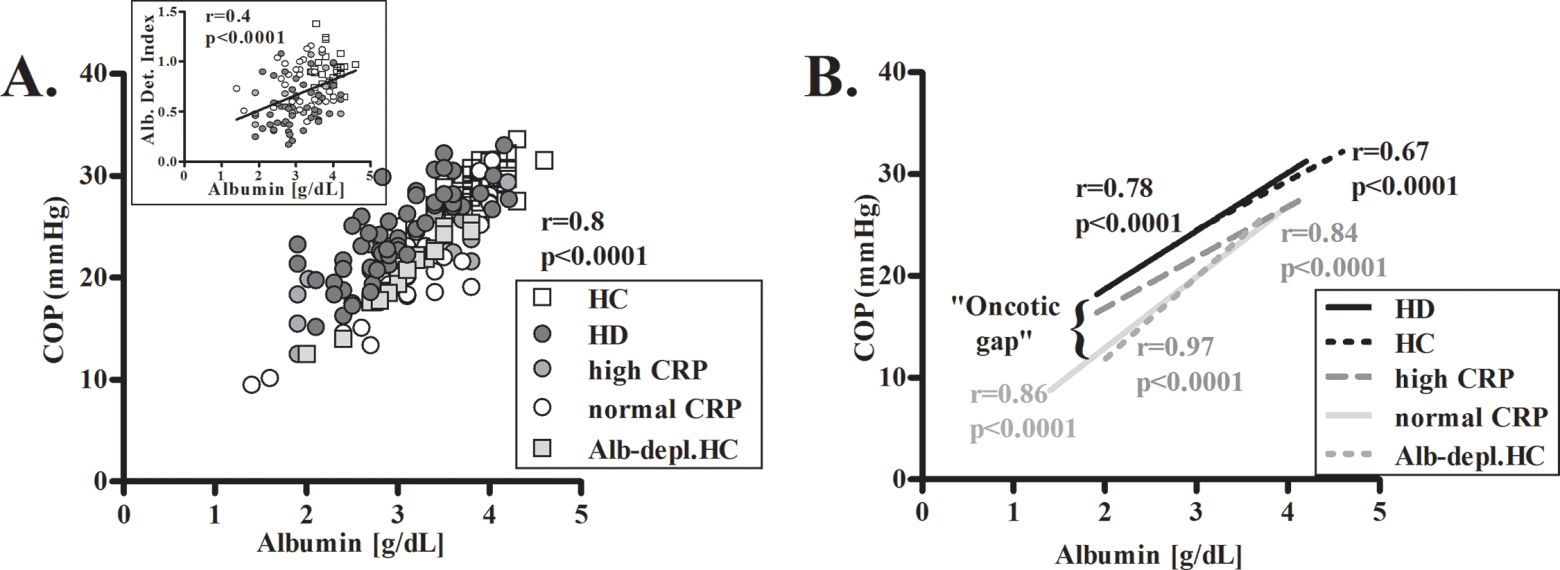
Albumin levels and COP values in the study groups. Sera samples from HC, proteinuria patients with low or high degree of inflammation (normal CRP. and high CRP, respectively), HD patients and HC sera after partial albumin depletion (Alb-depl.HC) were used for determination of albumin levels by BCG and measurements of COPCOP values were correlated with albumin levels measured by BCG in sera of all subjects **(A).** The separate regression lines are given for each subjects' group and for HC sera after partial albumin depletion (Alb-depl.HC) **(B)**. The BCG-measured albumin levels were also correlated with the albumin detection index (insert in Fig 1A).

Albumin levels were similar in proteinuric and HD groups but COP values in HD were significantly higher (Table 1). Albumin correlated with the index values of all subjects (Fig 1A insert). Albumin also correlated with COP when analyzed in all subjects, and in each group separately (Fig 1A and 1B). However, the regression lines in the HD group and in the proteinuria high inflammation group were clearly elevated compared to the regression lines in the proteinuria low inflammation and HC groups (Fig 1B). Partial depletion of albumin from HC sera, resulted in HC sera with decreased albumin levels, comprising a group of "hypoalbuminemic HC", which enabled the comparison of COP values with those of hypoalbuminemic patients (<3.8g/dl). The COP values in these "hypoalbuminemic HC" sera were as low as in the proteinuria groups (Fig 1B). When compared to these albumin-depleted HC, serum COP was 5.1 and 2.9 mmHg higher in the HD and proteinuria patients with inflammation respectively, comprising an "oncotic gap". In order to clarify the significance of these high COP values, and to examine the role of albumin modifications in this observation, two further types of experiments were performed: (a) COP measurements of *in-vitro* oxidized albumin; and (b) measurements of COP in albumin purified from patients' sera.

### COP of oxidized commercial human serum albumin

**Fig 2:**
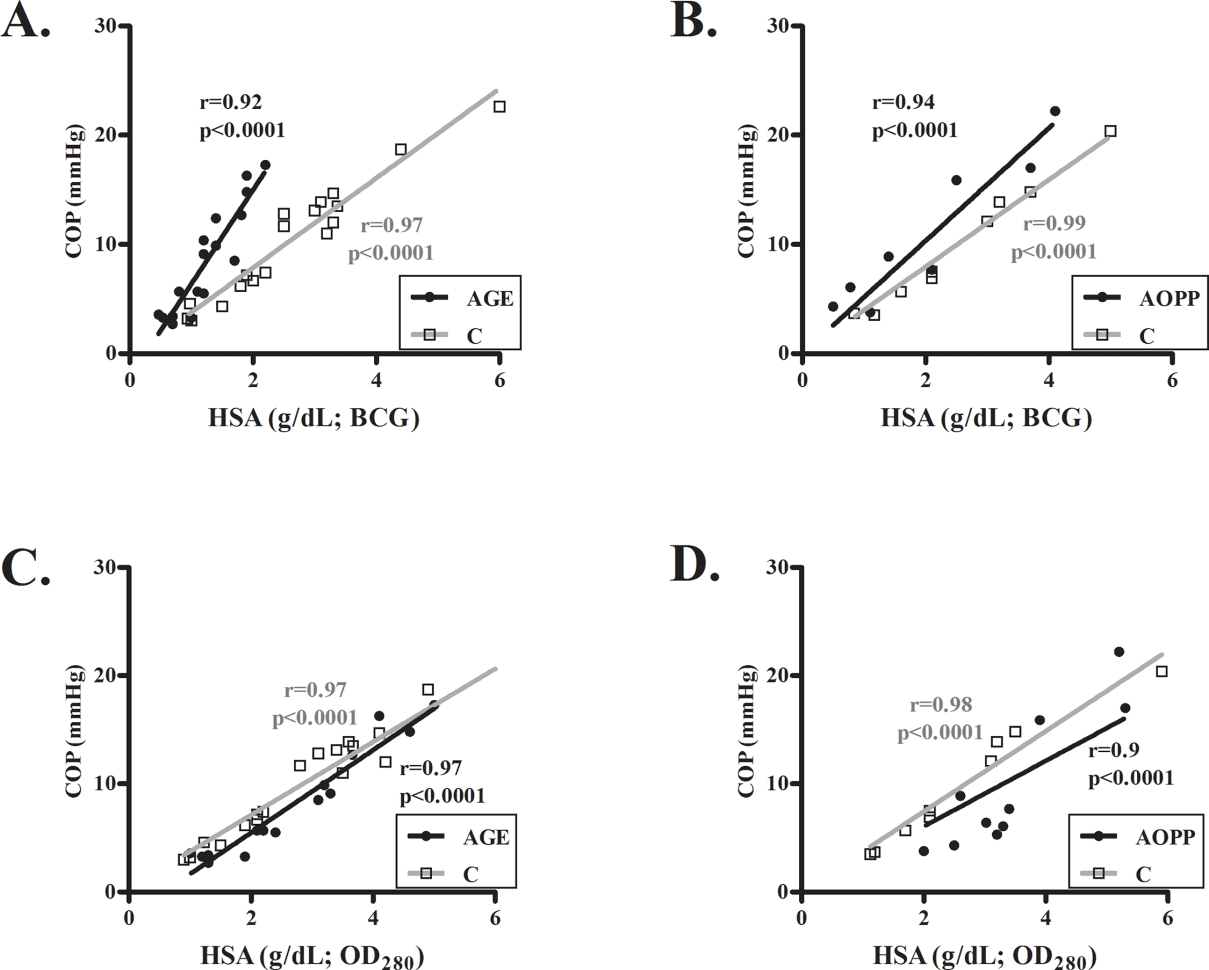
COP of in-vitro oxidized human serum albumin. Commercial HSA was glycoxidized **(A,C)** or oxidized by hypochlorite **(B,D)** *in-vitro* to produce AGE-albumin (AGE) and AOPP, respectively. COP values were correlated with albumin levels that were measured by BCG **(A,B)** or by OD_280_ **(C,D)** in the oxidized (●) and control (C;□) samples.

The COP values of glycoxidized or hypochlorite-modified HSA samples (purified commercial preparation), measured by BCG, were clearly higher than non-oxidized, control samples (Fig 2A and 2B). However, COP values were comparable when HSA levels were determined by OD_280_ (Fig 2C and 2D), an assay that is insensitive to albumin oxidation [4]. The *in-vitro* glycoxidation of HSA in these experiments was validated by color change (due to the Mailard "browning" reaction), evaluation of AGE content (by fluorescence) and determination of the albumin detection index, typically showing a 40–55% decrease vs. control samples [4].

### COP of purified albumin from subjects' sera

**Fig 3:**
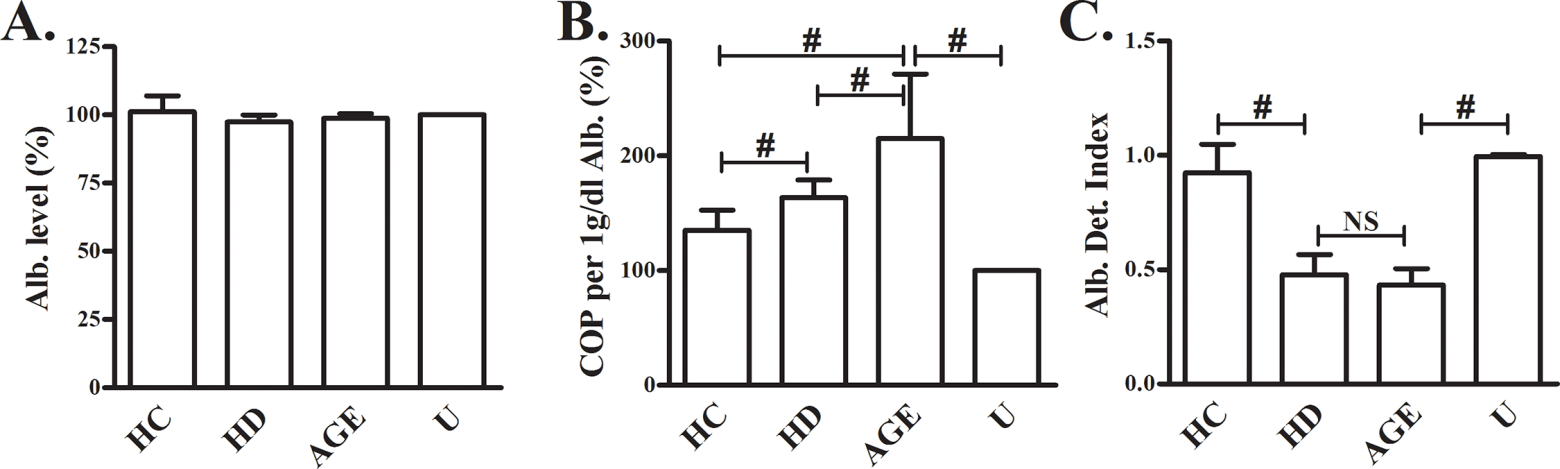
COP values of purified albumin from HC and HD sera. Albumin was purified from HD and HC sera, diluted to give equal concentrations and used for COP measurements after addition (of equal volume of each sample) to a normal (HC) sample with known albumin level and index (a single HC serum was used in each experiment). Albumin quantification by BCG (**A**), COP values (calculated per 1g/dl of the purified albumin and given as % of unmodified HSA) (**B**), and the albumin detection index values (**C**) are given as mean+SD. Glycoxidized (AGE) and unmodified (U) HSA that were treated identically, served as positive and negative controls, respectively. # indicates a significant p value (p<0.05). N = 5.

COP was measured indirectly in purified albumin from sera of HD and HC subjects, and calculated per 1g/dl albumin (BCG-measured). Although the measured albumin concentrations were identical (Fig 3A), COP values of purified albumin were 30% higher in HD compared to HC (Fig 3B, p = 0.03). COP values of AGE samples were significantly higher than any other sample. As expected, the albumin detection index showed an opposite tendency, namely, in the samples where COP values were the highest (AGE and HD) index values were decreased (Fig 3C). Index values were normal in the unmodified and HC albumin, in which COP values were low.

**Fig 4:**
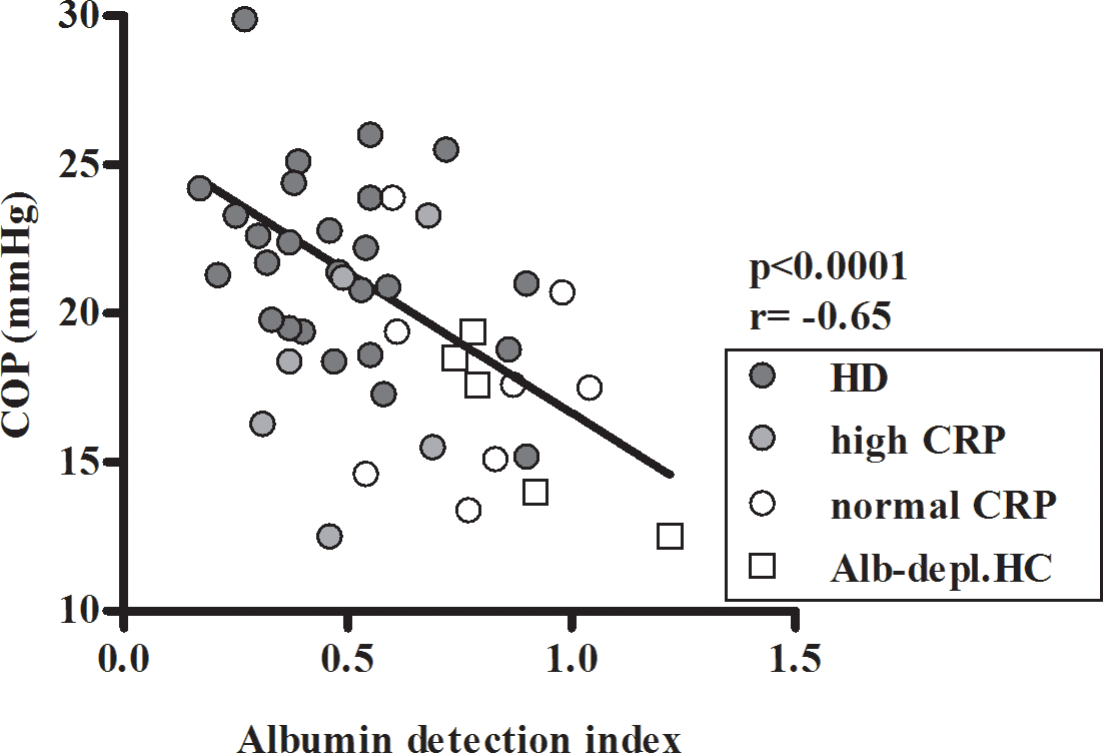
The association of COP with albumin detection index in sera. The association of COP (measured in serum) with albumin detection index was analyzed using selected subject's sera with albumin levels within a narrow range of 1.9–2.9 g/dl. HC sera with albumin levels within this range were obtained by partial albumin depletion (Alb-depl.HC).

As albumin oxidation influences COP, the possible inverse association between the index and COP values was examined. In order to dismiss the confounding effects of albumin levels on the analysis, the samples for this correlation included selected patients' sera and partially albumin-depleted HC sera, all with albumin levels of 1.9–2.9 g/dl. A significant inverse association was demonstrated between the index and the COP (Fig 4).

To investigate the potential contribution of other plasma proteins to the "oncotic gap" in HD patients, COP was measured in commercial preparations of purified human fibrinogen, albumin and immunoglobulins (IgG), in physiological concentrations. COP values of fibrinogen and IgG were significantly lower than albumin (Table 2). The calculated slopes of 0.001 and 1.26 indicate that an increased level of 100 mg/dl fibrinogen or 1 g/dl IgG would only raise COP value by 0.1 and 1.26 mmHg, respectively. Such potential increases in COP are considerably lower than the observed "oncotic gaps" of 5.1 mmHg observed in HD patients (Fig 1B).

**Table 2:** COP values of human serum albumin, fibrinogen and immunoglobulins.

	**COP increase (mmHg)**
**HSA (g/dL)**	**4.2**
**Fibrinogen (mg/dL)**	**0.001**
**IgG (g/dL)**	**1.26**

COP was measured in commercial, pure preparations of HSA, human fibrinogen and immunoglobulins (IgG), in their physiological range of concentrations. The given calculated slopes show the increase in COP for an increase in the protein concentration.

## Discussion

This study highlights the possibly misleading decrease in albumin concentrations, when measured by BCG, which complicates the interpretation of hypoalbuminemia in epidemiological and clinical studies. In patients with varying degrees of inflammation and oxidative stress such as proteinurics and patients on chronic HD therapy, the albumin detection efficacy of the BCG assay is greatly decreased. Consequently, the measured COP values are higher than expected for these "hypoalbuminemic" patients. The assumption that serum albumin levels are higher than measured was supported by COP measurements, used to indirectly assess albumin concentrations, in two types of experiments: in purified albumin from HD and HC subjects and in experiments where albumin was oxidized *in-vitro*.

It is postulated that the effect of "true" hypoalbuminemia on COP in acute illness is, at least in part, counterbalanced by an increase in the concentration of other plasma proteins, namely globulins and acute phase proteins [10]. Yet, their characteristics exclude any potential effect on the "oncotic gap" since the majority of these proteins are <30kDa, thus making them irrelevant to the COP measurements. The few proteins that are >30kDa, exist in low molar concentrations, 3–5 orders of magnitude lower than albumin. Although COP in this study was measured in serum, where fibrinogen (an established acute phase protein) is theoretically absent, the coagulation abnormalities which exist in HD patients [11] may lead to some residual fibrinogen in sera. However, even an increase of 100 mg/dl in fibrinogen concentration adds only 0.1 mmHg to the COP value, which is far below having any significance on the observed "oncotic gap". Globulins (140–170 kDa) are not acute phase proteins. However, in studies using mathematical calculations for assessment of COP, globulins have been suggested to play a role in maintaining COP [12]. We demonstrate that even a major increase of globulins, as shown in some clinical conditions [13], would add only 1.26 mmHg (for 1g/dl) to COP values. As patients with hyperglobulinemia were excluded from this study, there could not be a contribution of fibrinogen and IgG to the "oncotic gap". This data applies also to proteinuric patients with increased OS and inflammation, where the observed "oncotic gap" was ~3 mmHg.

Both OS and intracellular redox status are involved in inflammation and aging [14]. Hypoalbuminemia, inflammation and OS are biologically linked, as demonstrated by the correlations between the levels of inflammatory and OS biomarkers in hypoalbuminemic and normo-albuminemic HD patients [15]. This link is supported by the current study: the changes in the OS markers, AOPP and albumin detection index, are related to the degree of systemic inflammation (Table 1).

A potential weakness of the study is the lack of strict age-matched controls. To assess the possible confounding effects of age on the index and on the associated COP, we investigated the correlations between these parameters and age in our HC group. Age did not correlate with the index nor with COP (data not shown), thus ruling out age as a confounding factor. The existence of an "oncotic gap" between HD patients and proteinuric patients of similar ages further supports the notion that the oxidation of albumin, and not the subject's age, is a major contributor to this phenomenon.

The index appeared to exhibit some extent of inconsistency: purified HD-albumin and AGE-HSA samples had similar index values, but COP values for the later were significantly higher (Fig 3). This is explained by the differences in oxidative modifications between these samples. AGE-albumin is a single modification adduct, with a very dominant effect on the index [4]. In contrast, the purified HD-albumin contains various modifications such as AGEs, carbonyls, AOPP, oxidized thiols and other modifications [4,8,9,16–19], each with a different potential to decrease the index. Hence, although index values and the albumin concentrations appear to be similar, COP values may differ. The effect of oxidative modifications on albumin aggregation [20] may also influence COP measurements by decreasing the number of molecules, i.e. the molar concentrations of albumin. As for the clinical implication, in hypoalbuminemia with persistent OS, COP may be normal. This may be related to the absence of edema in these apparently hypoalbuminemic patients [21] and certainly deserves further investigation.

Hypoalbuminemia associated with large urinary losses of protein, which decreases albumin levels, may also be associated with albumin modification and variability in the measurements by BCG [7]. Consequently, other methods for differentiation of true and apparent hypoalbuminemia are required. Other clinical methods include bromocresol-purple (BCP) and immuno-nephelometry. These methods are based on binding a reagent to the albumin molecule, and are compromised by oxidative modifications [4]. As regards the immuno-nephelometry assay, which is considered to be the "gold standard" method for albumin measurements, our data indicates excellent correlation between results of BCG and the immuno-nephelometry (p<0.0001, supplementary data, Pannel A in S1 Fig). Moreover, a Bland-Altman analysis indicated a difference between BCG and immuno-nephelometry, that declines as albumin levels decrease, suggesting that these two methods show the highest similarity in the hypoalbuminemic range of albumin levels (supplementary data, Pannel B in S1 Fig). Methods such as optical density (OD_280_) and COP are insensitive to albumin oxidation, but are not albumin-specific, and the required albumin-purification steps limit their clinical use. Yet, COP may be used potentially as a tool for "differential diagnosis" between true hypoalbuminemia states that originate from decreased albumin production or albumin loss to those originating from decreased detection.

The rate of albumin synthesis is influenced by multiple factors including nutrition, inflammation, hormone status, and oncotic pressure. Increased COP decreases albumin gene expression [1,22], and in healthy animals is a primary determinant of albumin synthesis rate [23]. Therefore, the increased COP in HD patients (relative to HC) may have an inhibitory effect on albumin gene expression.

Numerous studies have pointed the importance of hypoalbuminemia in cardiovascular disease (CVD) of HD patients, suggesting hypoalbuminemia (BCG-measured) as a predictor of vascular morbidity and mortality [24]. Increased OS and high levels of oxidized/modified HSA contribute to cardiovascular complications [7,25–27] via various molecular mechanisms, such as control over the apolipoprotein B [28], cholesterol, and nitric oxide pool (our unpublished data) [29,30], effects on neutrophils [7] and endothel [26,27], and a general decrease in antioxidant capacity [3]. These mechanisms suggest oxidized albumin as a pathogenic factor with a potential to initiate and accelerate atherosclerosis.

In summary, this study illuminates the significant concentration of modified/oxidized serum albumin which leads to misinterpretation of albumin levels when the clinical laboratory assays BCG, BCP and immuno-nephelometry are used for quantification. The fact that albumin levels are systematically underestimated is shown by normal COP values. Serum albumin levels are commonly used for epidemiological studies, although without knowledge about the oxidative stress of the patients it is impossible to distinguish between true and apparent hypoalbuminemia. Since COP correlates with total serum albumin even when modified or oxidized, this measure has potential benefit in developing unique assays for improved assessment of true hypoalbuminemia.

## Supporting Information

S1 Fig Albumin levels measured by the BCG and the immune-nephelometry assays. Thirty three sera samples from HC, proteinuric patients and HD patients were used for determination of albumin levels by BCG and by the immuno-nephelometry assay (determined on BN ProSpec, SIEMENS), which is considered to be the "gold standard" method for albumin measurements. Part of the samples (20 out of 33) required a 25–50% dilution with saline prior to measurements, in order to achieve the minimal necessary volume for measurements. The correlation (**A**) between results of BCG and the immuno-nephelometry was significant (p<0.0001). The Bland-Altman analysis (**B**) indicated a difference between the results of these assays. This difference declines as albumin levels decrease, suggesting that the highest similarity between these methods is in the hypoalbuminemic range of albumin levels.
(DOC)
