# Supplementary material for: Unexpected Normal Colloid Osmotic Pressure in Clinical States with Low Serum Albumin
Source: PLoS One. 2016 Jul 25;11(7):e0159839. doi: 10.1371/journal.pone.0159839 (PMC4959682; doi:10.1371/journal.pone.0159839)
Supplement: S1 Fig — Thirty three sera samples from HC, proteinuric patients and HD patients were used for determination of albumin levels by BCG and by the immuno-nephelometry assay (determined on BN ProSpec, SIEMENS), which is considered to be the "gold standard" method for albumin measurements. Part of the samples (20 out of 33) required a 25–50% dilution with saline prior to measurements, in order to achieve the minimal necessary volume for measurements. The correlation (A) between results of BCG and the immuno-nephelometry was significant (p<0.0001). The Bland-Altman analysis (B) indicated a difference between the results of these assays. This difference declines as albumin levels decrease, suggesting that the highest similarity between these methods is in the hypoalbuminemic range of albumin levels. (DOC) [file pone.0159839.s001.doc]

**Supplementary data**


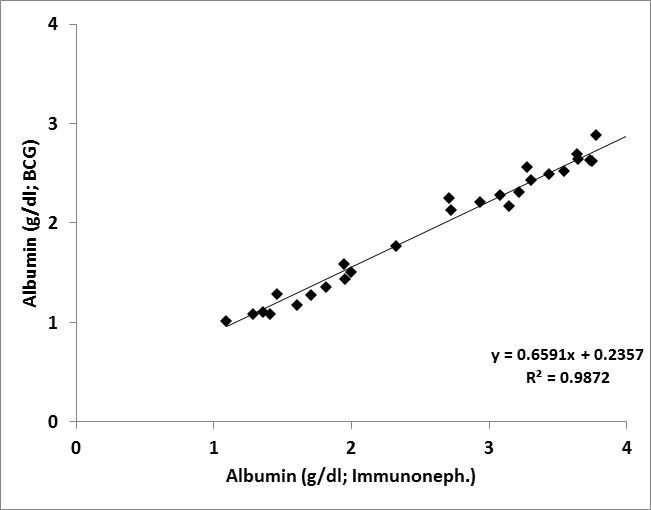
**A.**


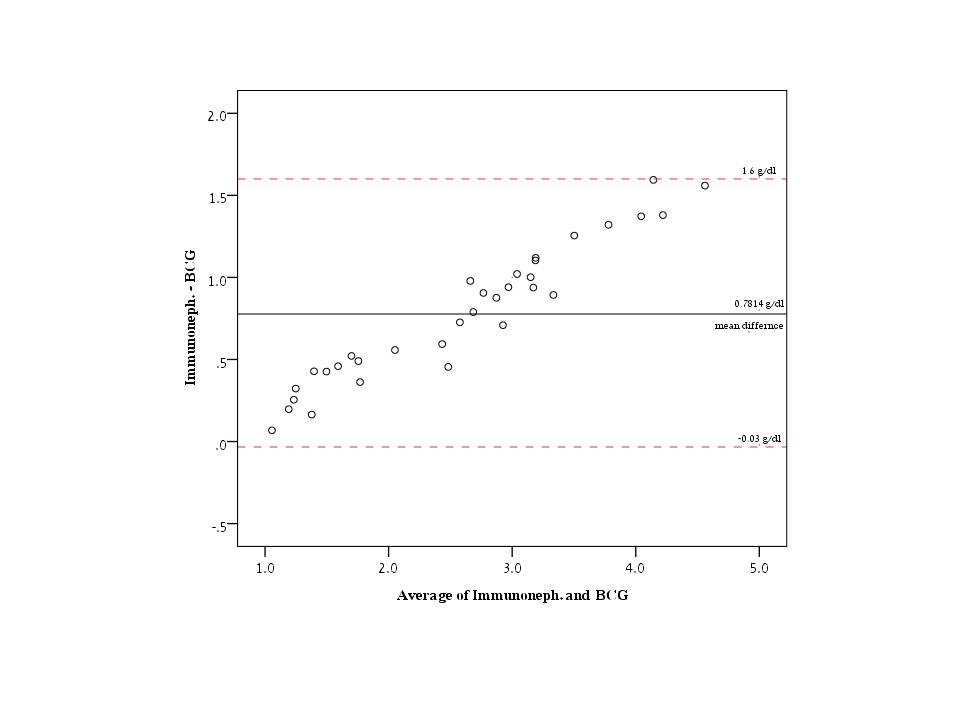
**B.**

**S1 Fig.** **Albumin levels measured by the BCG and the immune-nephelometry assays.**
